# Comparison of the results of four different refraction measurement devices in children with retinoscopy

**DOI:** 10.22336/rjo.2022.60

**Published:** 2022

**Authors:** Merve Simsek, Yesim Oral, Ozgur Erogul, Mehmet Cem Sabaner, Cem Simsek, Seray Yorukoglu

**Affiliations:** * Department of Ophthalmology, Mugla Education and Training Hospital, Mugla, Turkey; ** Department of Ophthalmology, Afyonkarahisar Health Sciences University, Afyonkarahisar, Turkey; *** Department of Ophthalmology, Mugla Sitki Kocman University Faculty of Medicine, Mugla, Turkey

**Keywords:** refraction, retinoscopy, amblyopia, spherical equivalent, autorefractometer

## Abstract

**Purpose:** The purpose of this study was to compare the results of 4 different autorefractometer devices with the results of retinoscopy in children.

**Methods:** A total of 120 eyes of 60 patients aged between 6 and 18, who applied to Afyonkarahisar Health Sciences University unit of Pediatric Ophthalmology, were included in the study. Refraction with Plusoptix A09 (photoscreener) without cycloplegia was the first to be measured. Spherical and cylindrical values were recorded. Then, half an hour after the patients were instilled 2 drops of cyclopentolate with an interval of 5 minutes, dilated retinoscopy was performed, and spherical and cylindrical values were recorded. Autorefractometer measurements with cycloplegia were performed with Canon RK-F1 autorefractometer, Nidek Tonoref III and Retinomax K-Plus 3, and spherical and cylindrical values were recorded.

**Results:** The mean age of the patients was 11.02 ± 2.1. The mean spherical equivalents were Canon RKF1 autorefractometer +0.045 ± 2.49, Nidek TonoRef III +0.023 ± 2.48, Retinomax K-Plus 3 +0.078 ± 1.42, Plusoptix A09 -0.119 ± 2.20, retinoscopy +0.124 ± 2.65. Moreover, the mean cylindrical values were Canon RK-F1 autorefractometer -0.893 ± 0.69, Nidek TonoRef III -0.927 ± 0.72, Retinomax K-Plus 3 -0.888 ± 0.73, Plusoptix A09 -0.883 ± 0.719, retinoscopy -0.923 ± 0.71. The statistical values compared with retinoscopy; Canon RKF1 spherical equivalent (p=0.376), cylindrical (p=0.515), Nidek TonoRef III spherical equivalent (p=0.485), cylindrical (p=0.198), Retinomax K-Plus 3 spherical equivalent (p=0.141), cylindrical (p=0.058), Plusoptix A09 spherical equivalent (p=0.085) and cylindrical (p=0.086) values were not different.

**Conclusions:** In spherical and cylindrical refractive error detection, all 4 devices showed reasonable and consistent results compared to retinoscopy.

## Introduction

Detection and correction of refractive errors in children is very important for the prevention of amblyopia and the elimination of visual disturbances [**1**]. The gold standard in the measurement of refractive error is sciascopic measurement after cycloplegia with 1% atropine sulfate [**2**]. However, 1% cyclopentolate hydrochloride drop is currently used more frequently in cycloplegia, as it has been shown to offer similar results with measurements after atropine and cycloplegia [**3**]. However, the use of sciascopy is limited, since it requires a lot of time in busy eye clinics. Several autorefractometer devices have been developed to overcome these limitations [**4**]. Plusoptix A09 (photoscreener) and Retinomax K-Plus 3 (handheld autorefractor) devices for younger children are also frequently used in clinics in addition to deskbound autorefractometer [**5**]. In this study, we aimed to compare the results of 4 different autorefractometer devices (Canon RKF1, TonoRef III, Retinomax K-Plus 3, Plusoptix A09) with the results of retinoscopy with cycloplegia in children.

## Material and Methods

Between January 2022 and February 2022, 120 eyes of 60 patients (34 were girls and 26 were boys) aged between 8-18 years (11.2 ± 2.1), who applied to Afyonkarahisar Health Sciences University unit of Pediatric Ophthalmology, were included in the study. The study was initiated after the approval of the hospital ethics committee. Refraction with Plusoptix A09 (photoscreener) without cycloplegia was the first to be measured. Spherical and cylindrical values were recorded. After half an hour of dripping cyclopentolate 2 times with a 5-minute interval, the refractive error was determined by subtracting +1.50D from the measurements made at the arm distance (67 cm) from the patient using the Heine Beta® 200 retinoscope (HEINE Ophthotecnic, Herrsching, Germany), hand-held trial lenses were used to neutralize the refractive error along the two principal meridians of each eye and spherical and cylindrical values were recorded. Retinoscopy was performed by two blind experienced ophthalmologists, and the mean of the measurements was planned to be used in the study. Autorefractometer measurements with cycloplegia were performed with Canon RK-F1 autorefractometer (Canon RKF1), Nidek TonoRef III (TonoRef III) and Retinomax K-Plus 3 (handheld autorefractor), and spherical and cylindrical values were recorded. The spherical equivalent was calculated by adding half of the cylindrical value to the spherical value. The best corrected visual acuities of the patients were calculated. Detailed anterior segment and posterior segment examination were performed. Patients with any ophthalmologic pathology other than refractive error were excluded from the study. Our data were analyzed by IBM SPSS Statistics for Windows, Version 22.0 software. The normality of data was confirmed by Shapiro-Wilk test. The results were compared using multivariate analysis of variance, linear regression, and the Pearson’s correlation analysis. The agreement between the devices was analyzed using the mean difference analysis and Bland-Altman analysis based on 95% limits of agreement (LoA). A p value of <0.05 was considered statistically significant. Reliability was analyzed using with the intraclass correlation coefficient (ICC), and its 95% confidence interval (CI). An ICC value of >0.8 was considered to indicate good repeatability, and a value >0.9 suggested excellent repeatability of measurements.

*Canon RK-F1 autorefractometer*

Canon RK-F1 (Canon Inc, Japan), one of the deskbound autorefractometer devices frequently used in clinics, has a microcomputer inside the device that extracts spherical, cylindrical lens refraction and then automatically displays this information corrected for a vertex distance of 12 mm. The device measures a spherical value between -30 and +20 Diopters (D), and cylindrical values up to 10 D [**6**].

*Nidek Tonorefractometer III*

Nidek Tonoref III (NIDEK Co, Ltd, Japan), another frequently used deskbound instrument in clinics, also has a built-in automatic refractometer, automatic keratometer, non-contact tonometer and pachymeter [**7**].

*Retinomax K-plus 3*

The refraction measurement is performed at approximately five centimeters from the children, with hand-held autorator Retinomax K-plus 3 (RTX; Right Mfg Co Ltd, Tokyo, Japan), which is not compatible with deskbound autoref devices and is preferred especially for young children. With Retinomax, monocular measurement is made, measuring refraction and keratometry, using a fogging mechanism to control accommodation. The spherical value range has a measuring range of -18 to +22 D, and 12 D for cylinders [**6**].

*PlusOptix A09*

PlusOptix A09 (Plusoptix GmbH, Nuremberg, Germany) is a non-invasive, binocular photorefractometer [**8**]. It is aimed to reduce the impact of accommodation by taking evaluations from 1 meter away. The measuring range starts from -7.00 diopters (D) for spherical and cylindrical values and has a measuring range of +5.00D [**8**].

## Results

The mean spherical equivalents were Canon RKF1 +0.045 ± 2.49, TonoRef III +0.023 ± 2.48, Retinomax K-Plus 3 +0.078 ± 1.42, Plusoptix A09 -0.119 ± 2.20, retinoscopy +0.124 ± 2.65. Also, the mean cylindrical values were Canon RKF1 -0.893 ± 0.69, Nidek TonoRef III -0.927 ± 0.72, Retinomax K-Plus 3 -0.888 ± 0.73, Plusoptix A09 -0.883 ± 0.719, retinoscopy -0.923 ± 0.71 (**Table 1**). 

**Table 1:** The descriptive of spherical and cylindrical values determined with five different methods

n: 120		Mean	Standard Deviation	Minimum	Maximum	P*
Canon	SPH	+0.045	2.49	-5.5	+8.0	SPH = 0.988 CYL = 0.947
	CYL	-0.893	0.69	-2.0	0.0	
Nidek	SPH	+0.023	2.48	-5.5	+7.5	
	CYL	-0.927	0.72	-2.25	0.0	
Retinomax	SPH	+0.078	1.42	-5.75	+7.25	
	CYL	-0.888	0.73	-2.50	0.0	
Plusoptix	SPH	-0.119	2.20	-5.75	+7.0	
	CYL	-0.883	0.719	-2.25	0.0	
Retinoscopy	SPH	+0.124	2.65	-5.5	+8.0	
	CYL	-0.923	0.71	-2.0	0.0	
*One-way ANOVA test result of five different methods. SPH = Spherical, CYL = Cylindrical						

The statistical values compared with retinoscopy Canon RKF1 spherical equivalent (p=0.376), Canon RKF1 cylindrical (p=0.515), TonoRef III spherical equivalent (p=0.485), TonoRef III cylindrical (p=0.198), Retinomax K-Plus 3 spherical equivalent (p=0.141), Retinomax K-Plus 3 cylindrical (p=0.058), Plusoptix A09 spherical equivalent (p=0.085), Plusoptix A09 cylindrical (p=0.086) values were not different (**Table 2**). 

**Table 2:** The results of Bland-Altman analyze the intraclass correlation coefficients calculated in comparison with retinoscopy for spherical and cylindrical values

n: 120				Mean difference					
		ICC	95% Confidence Interval	Mean	Standard deviation	Minimum	Maximum	95% LoA	P*
Canon	SPH	0.991	0.987 – 0.994	0.0229	0.283	-0.5	+1.0	-0.53 to +0.58	0.376
	CYL	0.964	0.948 – 0.975	0.0125	0.210	-0.75	+0.50	-0.40 to +0.42	0.515
Nidek	SPH	0.990	0.985 – 0.993	-0.0167	0.261	-0.50	+0.75	-0.53 to +0.49	0.485
	CYL	0.978	0.968 – 0.984	-0.0208	0.176	-0.75	+0.50	-0.37 to +0.32	0.198
Retinomax	SPH	0.957	0.938 – 0.969	-0.0417	0.308	-0.75	+0.50	-0.65 to +0.56	0.141
	CYL	0.976	0.966 – 0.984	0.0271	0.155	-0.50	+0.50	-0.28 to +0.33	0.058
Plusoptix	SPH	0.986	0.972 – 0.992	-0.0438	0.276	+0.50	+0.75	-0.58 to +0.50	0.085
	CYL	0.979	0.969 – 0.986	0.0229	0.145	-0.50	+0.50	-0.26 to +0.31	0.086
*Comparison of mean difference with retinoscopy (one-sample t test). SPH = Spherical, CYL = Cylindrical, ICC = Intraclass correlation coefficient, LoA = Limits of agreement									

Canon RKF1, Nidek TonoRef III, Retinomax K-Plus 3, Plusoptix A09 spherical equivalents showed high positive correlations (r=0.997), (r=0.997), (r=0.997), (r=0.997), respectively, when compared with retinoscopy (**Table 3**). 

**Table 3:** The results of correlation analysis performed for the **spherical** value of different methods

	Canon	Nidek	Retinomax	Plusoptix	Retinoscopy
Canon	–	–	–	–	–
Nidek	p<0.001 r=0.998	–	–	–	–
Retinomax	p<0.001 r=0.998	p<0.001 r=0.998	–	–	–
Plusoptix	p<0.001 r=0.996	p<0.001 r=0.996	p<0.001 r=0.996	–	–
Retinoscopy	p<0.001 r=0.997	p<0.001 r=0.997	p<0.001 r=0.997	p<0.001 r=0.997	–

When Canon RKF1, Nidek TonoRef III, Retinomax K-Plus 3, Plusoptix A09 cylindrical values were compared with retinoscopy, they showed high positive correlations (r=0.965), (0.978), (r=0.978), (r=0.981), respectively (**Table 4**).

**Table 4:** The results of correlation analysis performed for the **cylindrical** value of different methods

	Canon	Nidek	Retinomax	Plusoptix	Retinoscopy
Canon	–	–	–	–	–
Nidek	p<0.001 r=0.964	–	–	–	–
Retinomax	p<0.001 r=0.952	p<0.001 r=0.966	–	–	–
Plusoptix	p<0.001 r=0.964	p<0.001 r=0.975	p<0.001 r=0.980	–	–
Retinoscopy	p<0.001 r=0.965	p<0.001 r=0.978	p<0.001 r=0.978	p<0.001 r=0.981	–

**Fig. 1:**
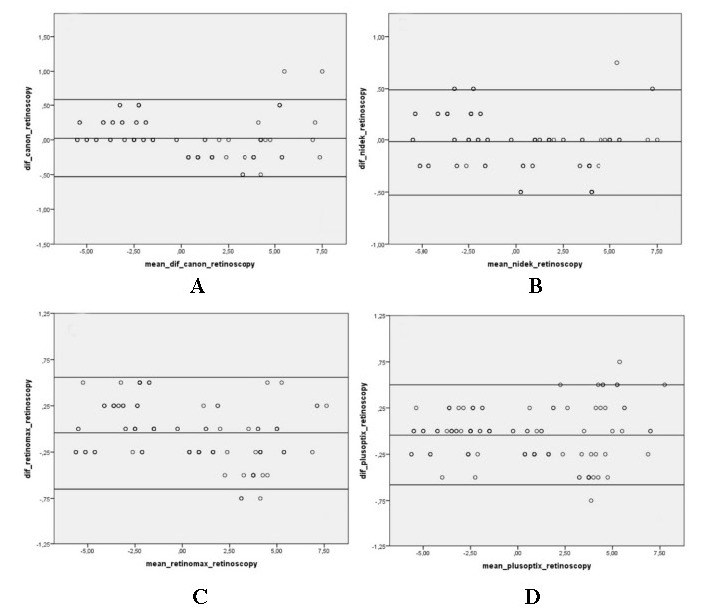
Bland-Altman plots showing comparison of spherical equivalent results between devices and retinoscopy. Y-axis: Difference and X-axis: The average of the tests compared, as well as the mean difference and ±1.96 axes are shown. **A.** Canon RK-F1, **B.** TonoRef III, **C.** Retinomax K- plus 3, **D.** PlusOptix A 09

**Fig. 2:**
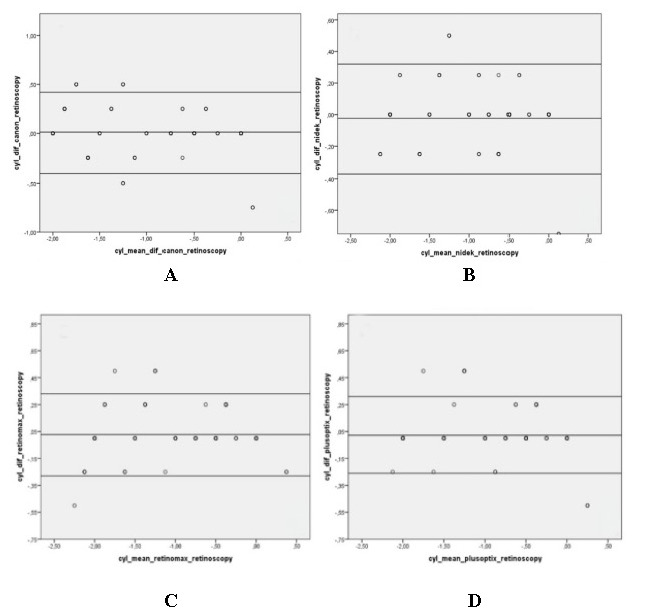
Bland-Altman plots showing comparison of cylindrical equivalent results between devices and retinoscopy. Y-axis: Difference and X-axis: The average of the tests compared, as well as the mean difference and ±1.96 axes are shown. **A.** Canon RK-F1, **B.** TonoRef III, **C.** Retinomax K- plus 3, **D.** PlusOptix A 09

Bland-Altman graphs showing the spherical equivalent results (**Fig. 1**) and cylindrical value results (**Fig. 2**) between the devices and retinoscopy also showed that the devices were compatible with retinoscopy in both spherical equivalent results and cylindrical value results.

## Discussion

The detection of refractive error in the pediatric age group is one of the most important factors in the detection of amblyopia, which can be treated in this age group [**9**]. Due to their high reproducibility and successful application by trained ordinary people, nurses and eye care professionals, autorefractors and vision screening are widely used in clinical practice and research settings to assess the refractive error status of children [**10**]. In this study, we compared the results of two deskbound autorefractometers (Canon RK-F1 vs. TonoRef III), a handheld autorefractometer (Retinomax K-Plus-3) and a photoscreener (PlusOptix A09) with the gold standard cycloplegic retinoscopy, the four devices being both spherical equivalent, helping us find cylindrical value results compatible with retinoscopy. Moreover, we were able to find that Canon RK-F1 cylindrical (p=0.515) and TonoRef III spherical equivalent (0.485) refractive errors were very close to retinoscopy. Retinomax K-Plus-3 cylindrical values were consistent with retinoscopy (p=0.058), but very close to being statistically different. PlusOptix A09 spherical (p=0.085) and cylindrical values (0.086) were also consistent with retinoscopy but very close to being statistically different. In a study by Luorno et al. in 2004, in which they compared SureSight handheld autorefractor measurements without cycloplegia with the Nidek AR-800 autorefractometer with cycloplegia and retinoscopy with cycloplegia, they found SureSight results to be more myopic [**11**]. In a study conducted by Bacher et al. in Germany in 2010, Retinomax K-Plus-3 results were found to be compatible with retinoscopy, but PlusOptix A09 results were not found to be compatible with retinoscopy [**12**]. Similarly, comparing PlusOptix S08 and Retinomax K-Plus-2 results with cycloplegic retinoscopy, Tamara et al. found in their study that both PlusOptixS08 and Retinomax K-Plus-2 were insufficient in detecting hyperopia [**13**]. In a large series study conducted in 2017, Ying et al. compared Grand Seiko autorefractor hand-held autorefractor and thought that hand-held autorefractor might be insufficient in determining hyperopia and astigmatism, since Granda Seiko gave higher spherical equivalent and cylindrical results compared to handheld autorefractor [**14**]. A similar result was found by Farook et al. in their study [**15**]. When comparing the results of handheld autorefractor and table-mounted autorefractor (Topcon RM8000B) with retinoscopy, hand-held autorefractor was not recommended for research [**15**]. However, in another study conducted in Singapore, hand-held autorefractor (Retinomax) was found to be compatible with Canon RK-F1 and retinoscopy, and was recommended for research [**16**]. Similarly, in our country, Tuncer et al. found the Retinomax hand-held autorefractor results to be compatible with the deskbound Nidek TonoRef III and retinoscopy [**17**]. Moreover, Yılmaz et al. found that PlusOptix A09 and Retinomax K-Plus-3 results in children were compatible and comparable with retinoscopy, and they stated that PlusOptix A09 could remove the necessity for cycloplegia in the measurement of refractive error in children [**8**]. In their study in which they compared the results of photoscreener, hand-held autorefractor, and deskbound device with retinoscopy with cycloplegia, Oral et al. found the results of three devices to be compatible with retinoscopy, similar to our study [**18**]. In a pilot study, conducted with preschool children in 2011, for the early diagnosis and prevention of amblyopia, Retinomax and Palm-Automatic Refractometer (Palm-AR) were found to be comparable to handheld autorefractor in terms of testability, sensitivity, and specificity [**19**]. Racano et al. compared 2Win and PlusOptix A12R devices with Retinomax and found that 2Win and PlusOptix A09 devices were compatible with each other, but insufficient in detecting hyperopia compared to the handheld autorefractor device [**20**]. However, in their study conducted in our country in 2021, Yalçınkaya et al. found the measurement results of two separate photoscreeners (SureSight and PlusOptix A09) to be compatible with retinoscopy [**21**].

## Conclusion

In our study, we also found spherical (p=0.085) and cylindrical values (0.086) of PlusOptix A09 to be compatible with retinoscopy, but very close to being statistically different. Therefore, we cannot state that PlusOptix A09 has not yet eliminated the necessity for cycloplegia in the measurement of refractive error in children. In conclusion, all four devices showed reasonable and consistent results in spherical and cylindrical refractive error detection compared to retinoscopy. 

**Conflict of Interest statement**

The authors state no conflict of interest. Both authors certify that they have no participation or association with any organization or individual, with any financial or non-financial interest in the subject matter or materials discussed in this article.

**Informed Consent and Human and Animal Rights statement**

All participants were verbally informed of the study before a written consent was obtained. In case of participants under 18 years old, a written consent was obtained from the parents.

**Authorization for the use of human subjects**

Ethical approval: The study method complied with the ethical principles laid down in the Helsinki Declaration and ethical approval was received from the ethics committee of Afyonkarahisar Health Sciences University, Afyonkarahisar, Turkey (2011-KAEK-2).

**Acknowledgements**

None.

**Sources of Funding**

This research did not receive any specific grant from funding agencies in the public, commercial, or non-profit sectors.

**Disclosures**

None.
